# Enhancing Hydration Stability and Proton Transport in Nafion/SiO_2_ Membranes for Medium- to High-Temperature PEMFCs

**DOI:** 10.3390/polym18030329

**Published:** 2026-01-26

**Authors:** Shuai Quan, Zheng Sun, Cong Feng, Lei Xing, Pingwen Ming

**Affiliations:** 1 School of Materials Science and Engineering, Tongji University, Shanghai 201804, China; 2 School of Chemistry & Chemical Engineering, University of Surrey, Guildford GU2 7XH, UK; 3 School of Automotive Studies, Tongji University, Shanghai 201804, China; pwming@tongji.edu.cn

**Keywords:** Nafion/SiO_2_ membranes, proton exchange membrane fuel cells, proton transport, hydration stability, hydrogen bonding

## Abstract

Perfluorosulfonic acid (PFSA) membranes suffer from severe conductivity decay caused by dehydration at elevated temperatures, hindering their application in medium- to high-temperature proton exchange membrane fuel cells (MHT-PEMFCs). To address this, Nafion/SiO_2_ composite membranes with systematically varied filler contents were fabricated via a sol–gel-assisted casting strategy to enhance hydration stability and proton transport. Spectroscopic and microscopic analyses reveal a homogeneous nanoscale dispersion of SiO_2_ within the Nafion matrix, along with strong interfacial hydrogen bonding between SiO_2_ and sulfonic acid groups. These interactions effectively suppress polymer crystallinity and stabilize hydrated ionic domains. Thermogravimetric analysis confirms markedly improved water retention in the composite membranes at intermediate temperatures. Proton conductivity measurements at 50% relative humidity (RH) identify the Nafion/SiO_2_-3 membrane as exhibiting optimal transport behavior, delivering the highest conductivity of 61.9 mS·cm^−1^ at 120 °C and significantly improved conductivity retention compared to Nafion 117. Furthermore, single-cell tests under MHT-PEMFC conditions (120 °C, 50% RH) demonstrate the practical efficacy of these membrane-level enhancements, with the Nafion/SiO_2_-3 membrane exhibiting an open-circuit voltage and peak power density 11.2% and 8.9% higher, respectively, than those of pristine Nafion under identical MEA fabrication and operating conditions. This study elucidates a clear structure–property–transport relationship in SiO_2_-reinforced PFSA membranes, demonstrating that controlled inorganic incorporation is a robust strategy for extending the operational temperature window of PFSA-based proton exchange membranes toward device-level applications.

## 1. Introduction

Proton exchange membrane fuel cells (PEMFCs) have garnered considerable attention as high-efficiency and environmentally friendly energy conversion systems, exhibiting strong potential for stationary power generation, portable electronics, and transportation applications [1,2,3,4,5]. Conventional low-temperature PEMFCs (<80 °C) have achieved significant commercialization; however, their operation remains limited by inherent temperature constraints [6]. These constraints include the strong hydration dependence of proton conduction, necessitating external humidification, the high CO sensitivity of Pt catalysts, which requires extensive fuel purification [7,8], and the generation of low-grade waste heat, which hampers the efficiency of combined heat and power (CHP) systems [9]. On the other hand, operating above 160 °C typically requires a substantial redesign of key fuel cell components, leading to increased system complexity and extended development cycles. Therefore, medium- to high-temperature operation (100–140 °C) is regarded as a practical compromise that balances performance enhancement with material compatibility.

Operating PEMFCs within the medium- to high-temperature (MHT) range offers several notable advantages. Elevated temperatures accelerate electrode reaction kinetics and significantly suppress CO adsorption on Pt surfaces, thereby enhancing tolerance to reformate fuels and simplifying fuel pretreatment requirements. Additionally, higher-grade waste heat improves overall CHP efficiency, while internal water management becomes less demanding due to reduced reliance on complex external humidification strategies [10,11]. Despite these advantages, achieving stable and efficient proton conduction under such conditions remains a critical challenge, primarily due to the performance limitations of current perfluorosulfonic acid (PFSA) membranes.

PFSA membranes predominantly rely on water-mediated proton transport and, therefore, suffer from dehydration, conductivity decay, and mechanical integrity degradation at temperatures above 100 °C under low-humidity conditions [12]. To address these limitations, various alternative membrane systems have been explored for high-temperature PEMFC applications. Sulfonated aromatic polymers, such as sulfonated poly(ether ether ketone) (SPEEK), have attracted significant attention due to their tunable ion exchange capacity and improved thermal stability compared to PFSA membranes; however, excessive sulfonation often leads to severe swelling, compromised mechanical strength, and reduced oxidative stability [13,14]. Ionic liquid (IL)-based membranes have also been developed to enable proton conduction under anhydrous or low-humidity conditions, benefiting from the intrinsic ionic conductivity and thermal stability of ILs [15], although issues related to ionic liquid leakage, phase separation, and long-term durability remain challenging. Polybenzimidazole (PBI)-based membranes doped with phosphoric acid have been widely regarded as state-of-the-art PEMFC electrolytes, capable of operating at high temperatures with minimal external humidification [16,17,18,19]. Nevertheless, their performance and durability are still limited by phosphoric acid leaching, catalyst poisoning, and relatively low power density, highlighting the continued need for alternative strategies to balance proton conductivity, mechanical integrity, and long-term stability under harsh operating conditions.

In light of these challenges, organic–inorganic composite membranes have emerged as a promising strategy for developing high-performance MHT proton exchange membranes (PEMs). The incorporation of inorganic nanofillers can enhance mechanical robustness, improve dimensional stability, and introduce additional proton-conducting pathways [20,21]. Among various fillers, SiO_2_ nanoparticles (SiO_2_ NPs) are particularly attractive for MHT-PEM applications due to their abundant surface silanol groups, which facilitate proton hopping and improve water retention under high-temperature, low-humidity conditions [22,23]. Moreover, their tunable surface area, porous structure, and versatile chemical functionalization (e.g., sulfonation, aminization, and phosphorylation) enable optimization of polymer–filler interfacial interactions and stabilization of proton-transport networks [24,25]. In addition, SiO_2_ NPs exhibit good compatibility with PFSA-based matrices and do not introduce electronic conductivity or undesirable electrochemical side reactions, which is critical for maintaining membrane integrity and fuel cell performance. Although other nanofillers, such as TiO_2_, graphene oxide, CuO, and Ag nanoparticles, have been reported to enhance membrane properties, their effectiveness under MHT operating conditions is often limited by particle agglomeration, interfacial incompatibility with PFSA matrices, and potential electronic conductivity. Moreover, inadequate filler dispersion and insufficient interfacial interactions can lead to performance deterioration, particularly under low-humidity conditions. Therefore, precise morphological control and interfacial engineering are crucial for fully exploiting the potential of SiO_2_-based composite membranes [26,27,28,29,30].

In our previous work [12], we systematically investigated the proton transport mechanisms of PFSA membranes under MHT conditions, revealing how microstructural evolution leads to conductivity decay. Leveraging these mechanistic insights, the present study aims to develop targeted material-level strategies to stabilize the membrane microstructure and enhance proton transport performance. To this end, Nafion/SiO_2_ composite membranes with varying filler loadings were fabricated via a sol–gel-assisted method and subjected to vacuum thermal treatment. Comprehensive physicochemical and microstructural characterizations were employed to elucidate the mechanisms underlying interfacial reinforcement. Proton conductivity was evaluated from 100 to 140 °C at 50% relative humidity (RH), alongside assessments of thermomechanical stability. Furthermore, single-cell tests were performed to validate the practical efficacy of the composite membranes. This study identifies the key structural and interfacial factors governing proton transport under MHT conditions, offering valuable guidelines for the rational design and optimization of next-generation composite PEMs.

## 2. Materials and Methods

### 2.1. Membrane Fabrication

A perfluorosulfonic acid ionomer dispersion (Nafion, 20 wt%, Chemours D2020) and tetraethyl orthosilicate (TEOS, Aldrich, Shanghai, China) were used as received. Anhydrous ethanol, deionized water, hydrochloric acid, and all other reagents were of analytical grade and used without further purification. A silica precursor sol was synthesized through an acid-catalyzed sol–gel procedure. TEOS, deionized water, and ethanol were mixed at a molar ratio of 0.05:0.2:1, and the pH of the mixture was adjusted to 2–3 using HCl to promote hydrolysis and condensation. The mixture was stirred thoroughly and aged for 24 h at room temperature, yielding a homogeneous silica sol composed of nanoscale SiO_2_ domains.

The aged sol was dispersed in deionized water and subsequently blended with a 20 wt% Nafion dispersion to prepare the composite casting solutions. The mixture remained stable without visible aggregation or phase separation during 2 h stirring at ambient conditions, ensuring a uniform distribution of SiO_2_ within the polymer matrix. The solutions were cast onto clean glass plates using a doctor blade with a controlled gap of 250 μm and dried in a vacuum oven at 80 °C for 2 h. After cooling to room temperature, the membranes were detached by immersing the glass substrates in water.

**Figure 1:**
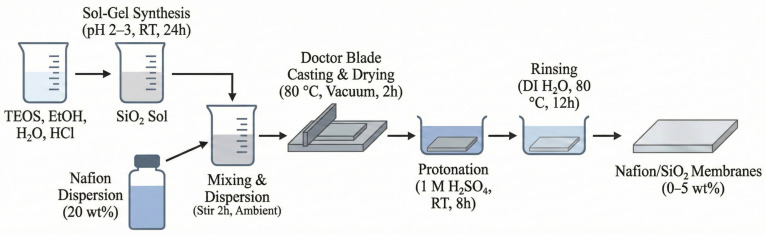
Schematic of the sol–gel-assisted membrane fabrication process.

Composite membranes containing 1–5 wt% SiO_2_ were fabricated by adjusting the silica sol content, and were denoted as Nafion/SiO_2_-1 to Nafion/SiO_2_-5. The SiO_2_ content varied from 1 to 5 wt.% to systematically evaluate the effect of filler loading on membrane morphology and performance, covering low, intermediate, and relatively high filler contents commonly reported for organic–inorganic composite PEMs. A pristine Nafion membrane (0 wt% SiO_2_), denoted as Nafion/SiO_2_-0, was also prepared using the same procedure as the composite membranes and served as the reference sample. All membranes underwent protonation and post-treatment by immersion in 1 M H_2_SO_4_ at room temperature for 8 h, followed by thorough rinsing in deionized water at 80 °C for 12 h to remove residual organics and ensure complete protonation of the sulfonic groups. The dried membranes exhibited final thicknesses in the range of 85–105 μm, with only slight variations depending on the SiO_2_ content. The slightly increased thickness observed for Nafion/SiO_2_-5 may be attributed to the higher solid content of the casting solution. The compositions of the prepared membranes are summarized in Table 1. A schematic illustration of the sol–gel-assisted membrane fabrication process is provided in Figure 1.

### 2.2. Physicochemical and Morphological Characterization

The microstructure and surface morphology of the Nafion/SiO_2_ composite membranes were characterized by an atomic force microscope (AFM, FastScan Bio, Bruker, Billerica, MA, USA) operated in modulus imaging mode, which was employed to evaluate the size and dispersion of SiO_2_ particles within the polymer matrix. The elemental composition and chemical states of the composite membranes were analyzed using X-ray photoelectron spectroscopy (XPS, ESCALAB 250Xi, Thermo Fisher Scientific, Waltham, MA, USA). In addition, the chemical structure and intermolecular interactions of the membranes with different SiO_2_ contents were investigated by Fourier transform infrared (FTIR) spectroscopy (INVENIO, Bruker, Karlsruhe, Germany) over the wavenumber range of 4000–400 cm^−1^.

The crystallinity and structural evolution of the membranes at elevated temperatures were monitored by in situ X-ray diffraction (XRD, DX-2700BH, Dandong Haoyuan Instrument Co., Ltd., Liaoning, China). Thermal stability and water uptake behavior were evaluated using a simultaneous thermal analyzer (STA 2500 Regulus, Netzsch, Selb, Germany), which simultaneously recorded thermogravimetric analysis (TGA) signals. Tensile mechanical properties were measured at room temperature using a dynamic mechanical analyzer (DMA Q850, TA Instruments, New Castle, DE, USA) operated in tensile mode. The tests were conducted at a constant strain rate of 2% min^−1^, and engineering stress–strain curves were recorded until sample fracture or until the instrument’s maximum allowable strain was reached.

In-plane proton conductivity was measured using a four-probe membrane test system (MTS 740, Scribner Associates Inc., Southern Pines, NC, USA). Electrochemical impedance spectroscopy (EIS) measurements were performed under controlled temperature and relative humidity conditions, with nitrogen used as the purge gas at a flow rate of 500 sccm. Membrane resistance was determined from the high-frequency intercept of the Nyquist plot at 20 MHz. Prior to each measurement, membranes were heated to the target temperature and equilibrated for 30 min to ensure thermal and humidity stability. Proton conductivity measurements were carried out with three consecutive impedance scans recorded at each temperature to ensure reproducibility. All samples were tested at a constant relative humidity of 50% RH, while the temperature varied from 80 to 140 °C in 10 °C increments. Proton conductivity was calculated based on the measured membrane resistance, sample dimensions, and electrode spacing. Measurements were conducted under controlled low-humidity conditions relevant to PFSA-based membranes, where water-mediated proton transport remains operative.

### 2.3. MEA Fabrication and Single-Cell Testing

Catalyst inks were prepared by mixing a 50 wt% Pt/C catalyst, Nafion ionomer solution (10 wt% solids), isopropanol, and deionized water, followed by ultrasonication for 30 min to obtain a homogeneous dispersion. The inks were deposited onto the proton exchange membrane by manual air-spray coating using a handheld spray gun to fabricate catalyst-coated membranes (CCMs). The coated membranes were dried at 80 °C for 30 min. The platinum loadings were 0.4 mg_Pt cm^−2^ at both the anode and the cathode.

The CCMs were framed with 25 μmthick poly(ethylene naphthalate) gaskets and hot-pressed at 130 °C under 0.6 MPa for 180 s. Gas diffusion layers (GDLs, 235 μm thick) were placed on both sides of the CCM to assemble membrane electrode assemblies (MEAs) with an active area of 25 cm^2^. The MEAs were mounted in single cells equipped with serpentine flow fields and evaluated using a commercial fuel cell test station. Prior to performance measurements, the cell was conditioned at the target operating temperature by holding the cell voltage at 0.6 V for 2 h.

Polarization and power density curves were recorded at a back pressure of 200 kPa (gauge), with hydrogen supplied to the anode and air to the cathode at fixed flow rates of 1 standard liter per minute (slm) and 2 slm, respectively. The inlet humidification was controlled to achieve a target RH of 50% at each operating temperature by adjusting the dew point (63.8 °C at 80 °C, 81.7 °C at 100 °C, and 99.5 °C at 120 °C). Polarization curves were obtained by continuous linear current scanning, and the cell voltage was logged every 30 s. All voltages reported are non-iR-corrected.

## 3. Results and Discussion

### 3.1. Structural Incorporation and Interfacial Interactions of SiO_2_ in Nafion

**Figure 2:**
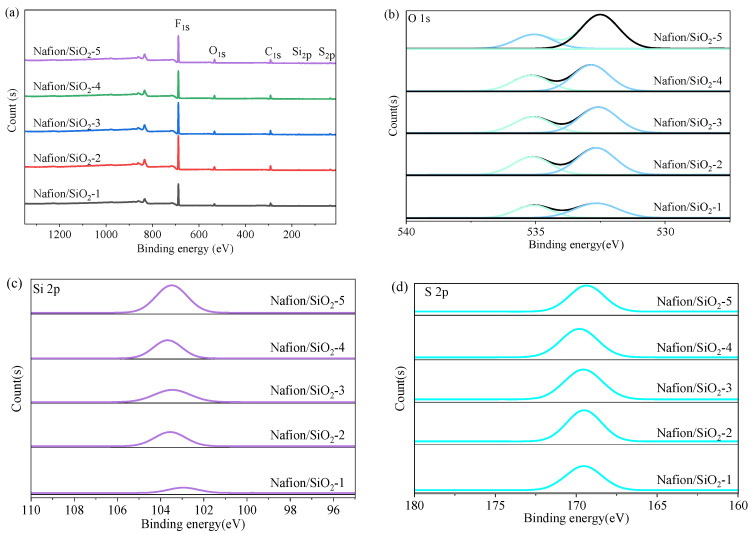
XPS characterization of Nafion/SiO_2_ composites: (**a**) survey spectra; (**b**) O 1s; (**c**) Si 2p; (**d**) S 2p high-resolution spectra.

Nanostructured SiO_2_ was incorporated into Nafion through a controlled synthesis route. The surface hydroxyl groups of SiO_2_ interact with the terminal –SO_3_H groups of Nafion via hydrogen bonding, providing potential pathways for proton hopping, particularly under low humidity. XPS was used to probe the chemical environments of O, Si, and S (Figure 2a). The O 1s spectra (Figure 2b) consist of two components at 532.5 and 535.2 eV. The former corresponds to lattice oxygen in Si–O–Si and S=O groups, while the latter arises from surface hydroxyls and adsorbed water associated with SiO_2_ and the hydrated membrane. The increasing intensity of the hydroxyl/water component up to 4 wt% SiO_2_ indicates a progressively strengthened hydrogen-bonding network, followed by a decrease at 5 wt%, suggesting saturation and partial disruption at excessive filler loadings. The Si 2p peak at 103.6 eV (Figure 2c) confirms the presence of Si^4+^ in tetrahedrally coordinated SiO_2_, indicating successful incorporation without altering silica’s chemical structure. The S 2p peak at 169.5 eV (Figure 2d) verifies that the sulfonate groups remain chemically intact and that the oxidation state of sulfur is unaffected. Elemental ratios derived from XPS match the nominal compositions, validating the designed SiO_2_ loadings.

In addition to the intensity evolution, slight shifts in the binding energies of the Si 2p and S 2p peaks are observed with increasing SiO_2_ content. For SiO_2_ loadings up to 4 wt%, the gradual increase in binding energy suggests strengthened interfacial interactions between SiO_2_ nanoparticles and the PFSA matrix, likely arising from enhanced hydrogen bonding and local electronic interactions. At a higher loading of 5 wt%, a slight decrease in binding energy is observed, which may be associated with saturation of interfacial interactions and partial disruption of the hydrogen-bonding network due to excessive filler content. These trends indicate that interfacial effects between SiO_2_ and sulfonic acid groups are maximized at intermediate filler loadings.

### 3.2. Surface Morphology and Nanomechanical Properties of Composite Membranes

**Figure 3:**
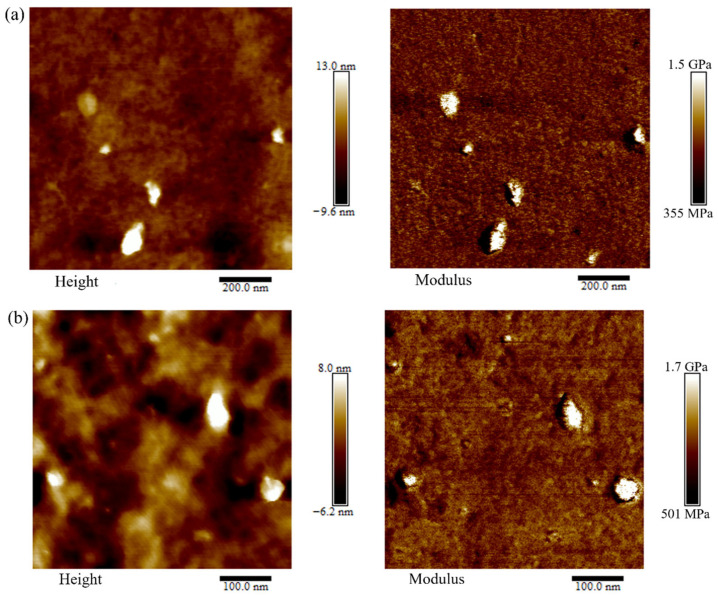
AFM height and modulus images of Nafion/SiO_2_ composite membranes with (**a**) 2 wt% and (**b**) 3 wt% SiO_2_.

AFM was employed to probe the nanoscale morphology and mechanical heterogeneity of Nafion/SiO_2_ composite membranes containing 1–5 wt% SiO_2_. Large-area AFM height images were collected to evaluate surface dispersion and possible nanoparticle agglomeration at length scales comparable to SEM observations (Figures S1 and S2), which demonstrates that, across the entire composition range, SiO_2_ nanoparticles are homogeneously embedded within the Nafion matrix without evidence of macroscopic phase separation. Representative height and modulus maps are shown in Figure 3. The height images reveal discrete nanoscale protrusions that are uniformly distributed over the membrane surface, indicating effective confinement of the inorganic phase within the polymer network. The lateral dimensions of these features are predominantly in the range of ~10–100 nm, suggesting that SiO_2_ remains well dispersed at the nanoscale even at higher filler loadings. As the SiO_2_ content increases from 1 to 5 wt%, the number density of SiO_2_-rich domains gradually increases, accompanied by a modest rise in surface roughness. Nevertheless, the overall morphology remains continuous and compact, implying strong interfacial interactions between SiO_2_ nanoparticles and the Nafion matrix that suppress large-scale aggregation. In addition to the AFM height images, the surface roughness of the membranes was quantitatively analyzed in terms of the average roughness (Ra) and root mean square roughness (Rq). The corresponding values for all membranes are summarized in Table S1. The results indicate that the surface roughness remains at the nanometer scale, with only moderate variations upon the incorporation of SiO_2_ nanoparticles.

The corresponding modulus maps exhibit pronounced mechanical contrast between the polymer-rich regions and SiO_2_-rich domains. The latter display elastic moduli in the range of approximately 1.2–1.7 GPa, substantially higher than that of the surrounding Nafion matrix. With increasing SiO_2_ content, high-modulus regions become more prevalent and spatially interconnected, reflecting the progressive formation of a nanoscale reinforcing framework within the membrane. Notably, despite the enhanced stiffness, the mechanical landscape remains relatively uniform across the membrane surface, indicating that the incorporation of SiO_2_ nanoparticles strengthens the membrane without introducing severe local mechanical heterogeneity. These observations demonstrate that SiO_2_ can be efficiently integrated into the Nafion matrix over a broad composition window, yielding well-dispersed nanoscale domains that provide mechanical reinforcement while preserving structural continuity.

Collectively, the XPS and AFM results indicate that SiO_2_ nanoparticles are homogeneously distributed within the Nafion matrix with strong interfacial compatibility. Such a well-integrated nanoscale inorganic phase is expected to play a key role in governing the transport properties of the composite membranes, as discussed in the following sections.

### 3.3. Influence of SiO_2_ Loading on the Molecular Structure of Nafion

**Figure 4:**
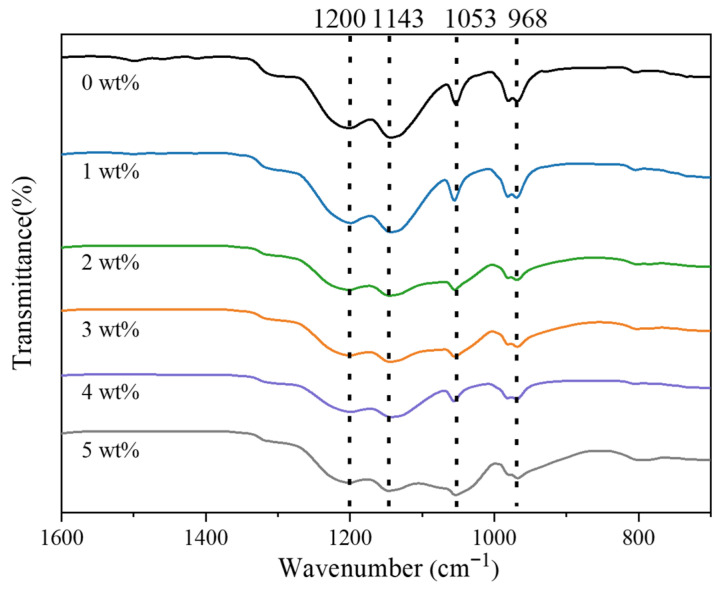
FTIR spectra of Nafion/SiO_2_ composite membranes with different SiO_2_ contents.

FTIR spectroscopy was used to assess how SiO_2_ incorporation alters the molecular environment of Nafion (Figure 4). Membranes containing 0–5 wt% SiO_2_ show systematic spectral changes. The vibrational feature at ~1200 cm^−1^, arising from asymmetric CF_2_ stretching in the perfluorinated backbone, shifts slightly to lower wavenumbers as the SiO_2_ content increases. This red shift, together with peak broadening and reduced intensity, signifies a disturbance of chain packing and a reduction in the crystallinity of the perfluorosulfonic domains. The ~1145 cm^−1^ symmetric CF_2_ stretching band shows a minor blue shift but retains its spectral shape, suggesting that the C–F backbone remains structurally intact, while weak interfacial interactions with SiO_2_ slightly stiffen the CF_2_ environment. A stronger response emerges at ~1053 cm^−1^, where the SO_3_^−^ symmetric stretch intensifies and shifts to lower wavenumbers. The trend signifies reinforced hydrogen bonding between Si–OH and sulfonic groups, enhanced interfacial hydration, and contributions from Si–O–Si vibrations—factors that collectively alter S–O bond energies and amplify local polarity. The ~968 cm^−1^ C–O–C ether mode also moves slightly to lower wavenumbers, with a moderate rise in intensity. This shift reflects SiO_2_-induced modifications in the side-chain environment, driven by hydrogen bonding, interfacial hydration, and possibly weak Si–O–C interactions.

Overall, the FTIR trends confirm that SiO_2_ perturbs local packing and hydration around sulfonic and ether groups while preserving the integrity of the Nafion backbone. The strengthened hydrogen-bonding network and increased density of hydrophilic sites are expected to enhance water retention and proton transport at appropriate SiO_2_ loadings.

### 3.4. Structural Disorder and Crystallinity Suppression

**Figure 5:**
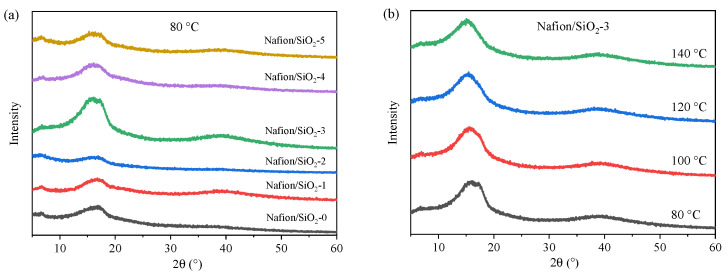
XRD patterns of Nafion/SiO_2_ composite membranes. (**a**) Different SiO_2_ contents at 80 °C, (**b**) 3 wt% SiO_2_ at different temperatures.

Figure S3 summarizes the XRD profiles of the Nafion/SiO_2_ membranes under varying filler loadings and temperatures. All samples exhibit broad reflections at ~17° and ~38°, characteristic of the semi-crystalline fluorocarbon backbone and the short-range intrachain order. At a test temperature of 80 °C, increasing the SiO_2_ loading progressively suppresses the ~17° diffraction peak and induces marked peak broadening, signaling a continuous reduction in crystalline domain size and long-range order (Figure 5a). Even at 1 wt% loading, interfacial hydrogen bonding between Si–OH and –SO_3_H groups disrupts chain stacking and partitions the crystalline regions into smaller, less coherent lamellae. Higher loadings (4–5 wt%) accentuate this effect, as steric constraints and interfacial interactions inhibit lamellar packing and drive the system toward an increasingly amorphous configuration. A parallel decrease in the ~38° feature confirms the loss of short-range intrachain order. Notably, the Nafion/SiO_2_-3 membrane exhibits a distinct evolution of the diffraction profile compared with other compositions, particularly in the broad peak region around 17°. This behavior, commonly associated with the semi-crystalline organization of PFSA chains [31], suggests an optimized microphase structure at intermediate SiO_2_ loading, where polymer–filler interactions promote a more favorable balance between ordered and disordered domains.

Thermal effects follow a similar trend (Figure 5b). Raising the measurement temperature from 80 °C to 140 °C causes the ~17° reflection of the unfilled membrane to shift to lower angles and diminish in intensity, consistent with thermal expansion, enhanced chain mobility, and the collapse of semi-crystalline lamellae. In contrast, SiO_2_-containing membranes exhibit significantly smaller peak shifts and reduced intensity loss, indicating that the inorganic domains act as physical constraints that suppress thermal relaxation and stabilize the polymer microstructure (Figure S3). Under the combined influence of high temperature and high SiO_2_ loading (5 wt%–140 °C), both diffraction features show pronounced attenuation and broadening, leaving only weak residual short-range order (Figure S3c). This response demonstrates the cooperative nature of thermal activation and interfacial disruption, which jointly promote chain disorder and drive the transition from a semi-crystalline to a largely amorphous state.

### 3.5. Mechanical Response of SiO_2_–Nafion Composite Membranes

**Figure 6:**
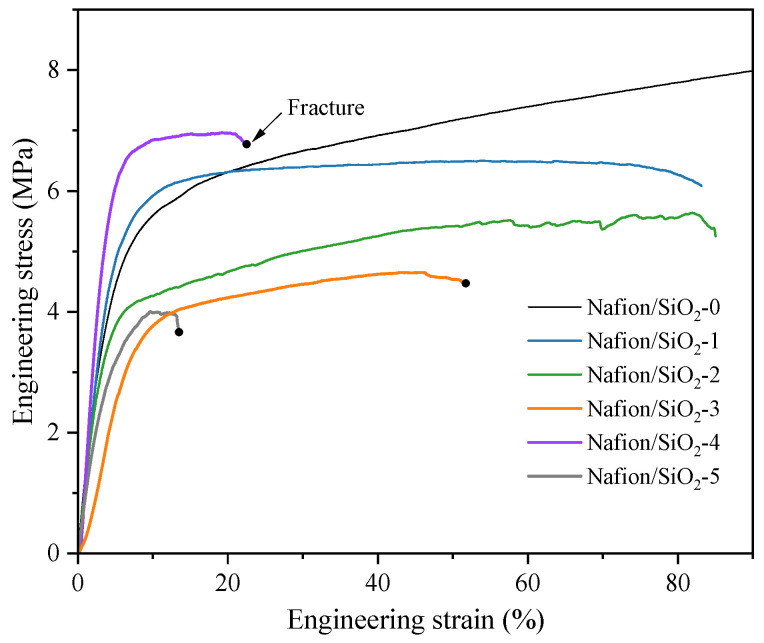
Engineering stress–strain curves of Nafion membranes containing different SiO_2_ contents obtained from DMA tensile tests. Black dots mark the initiation of catastrophic fracture. Curves without markers were truncated at the fracture onset.

Figure 6 presents the engineering stress–strain responses of Nafion membranes incorporating different SiO_2_ contents under DMA tensile loading. Neat Nafion exhibits a highly ductile mechanical behavior, characterized by a continuous stress increase with strain and the absence of fracture within the maximum strain range accessible by the instrument, indicating that its intrinsic ductility exceeds the experimental deformation window.

The incorporation of low SiO_2_ contents (1–2 wt%) leads to a moderate reduction in ductility while largely preserving the overall stress level during plastic deformation. Although these samples fracture within the tested strain range, the stress–strain curves are plotted only up to the onset of fracture and are not extended beyond this point. This behavior suggests that limited filler addition restricts polymer chain mobility without significantly disrupting the structural continuity of the Nafion matrix.

As the SiO_2_ content increases to 3 wt%, a pronounced decrease in elongation at break is observed, accompanied by a reduction in maximum stress, marking the onset of ductility loss and the increasing influence of filler–matrix interactions and localized stress concentrations. A distinct transition in mechanical behavior occurs at 4 wt% SiO_2_, where the membrane exhibits a substantially increased initial stiffness, as reflected by the steep initial slope of the stress–strain curve. This stiffness enhancement is, however, coupled with a sharp reduction in elongation at break and an abrupt stress drop at failure, indicative of brittle fracture. Such behavior suggests the formation of a rigid, filler-dominated network at this critical loading, which effectively suppresses large-scale polymer chain rearrangement but simultaneously compromises the membrane’s ability to accommodate tensile deformation.

Further increasing the SiO_2_ content to 5 wt% does not result in additional stiffness enhancement. Instead, both the initial stiffness and tensile strength deteriorate markedly, together with severely reduced deformability. This degradation is attributed to excessive filler loading, which promotes SiO_2_ agglomeration and disrupts the continuity of the polymer matrix, leading to inefficient stress transfer and premature failure.

Overall, these results reveal a clear stiffness–ductility trade-off induced by SiO_2_ incorporation in Nafion membranes, identifying 4 wt% SiO_2_ as a critical threshold for mechanical reinforcement, beyond which further filler addition becomes detrimental to mechanical integrity. This conclusion is consistent with the XPS-based prediction.

### 3.6. Thermogravimetric Behavior and Water Retention

**Figure 7:**
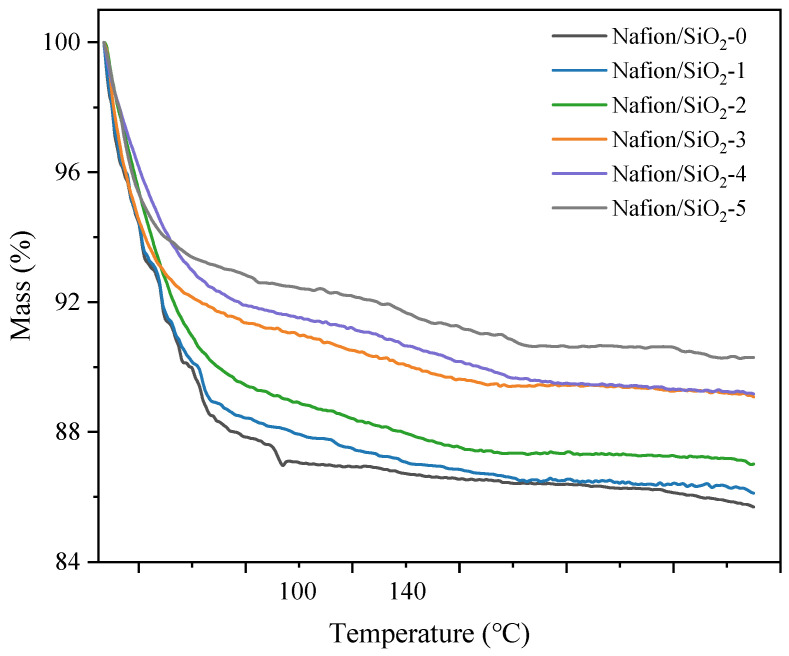
Thermogravimetric behavior of Nafion and Nafion/SiO_2_ composite membranes.

The thermal behavior of pristine Nafion and the composite membranes containing 1–5 wt% SiO_2_ was evaluated by TGA in the range of 25–270 °C (Figure 7). TGA measurements were conducted up to 270 °C to evaluate membrane thermal stability well above the operating temperature range of MHT-PEMFCs. All membranes exhibit a progressive mass loss with increasing temperature, which is predominantly associated with the removal of free and weakly bound water. Notably, the total mass loss decreases systematically with increasing SiO_2_ content, indicating enhanced water retention and improved thermal stability induced by the inorganic phase.

Below 100 °C, pristine Nafion undergoes rapid dehydration, reflecting a high fraction of loosely bound water. In contrast, the composite membranes display a more gradual mass decrease, suggesting strengthened water–polymer interactions upon SiO_2_ incorporation. This stabilization effect becomes more pronounced in the MHT range (100–140 °C), which is particularly relevant for elevated-temperature membrane operation. In this regime, the composite membranes retain substantially higher residual mass than pristine Nafion, consistent with improved water retention and structural integrity.

The enhanced thermal stability is attributed to interfacial hydrogen bonding between Si–OH groups, sulfonic acid moieties, and water molecules, together with microstructural constraints imposed by the dispersed inorganic phase that limit polymer chain mobility and stabilize the hydrated ionic domains. At temperatures above 140 °C, the mass loss rate decreases for all samples, indicating depletion of removable water and the dominance of the polymer backbone and thermally stable inorganic components.

The corresponding derivative thermogravimetric (DTG) curves further confirm that no pronounced degradation peaks are observed up to 270 °C, indicating sufficient thermal stability within the investigated temperature range (Figure S4). Collectively, the improved water retention and thermal stability conferred by SiO_2_ incorporation provide a structural foundation for the temperature-dependent proton conductivity behavior discussed in the following section.

### 3.7. Proton Conductivity

**Figure 8:**
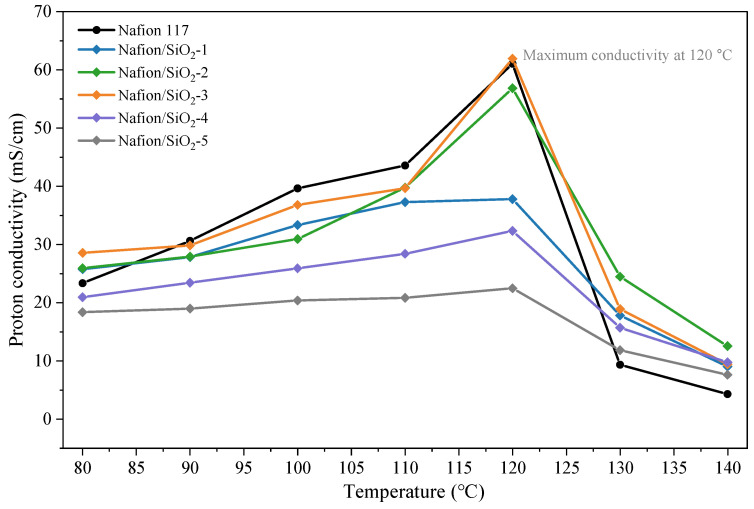
Proton conductivity of Nafion 117 and Nafion/SiO_2_ composite membranes as a function of temperature.

The proton conductivity of pristine Nafion and Nafion/SiO_2_ composite membranes was evaluated in the temperature range of 80–140 °C at 50% RH, as shown in Figure 8. For all membranes, proton conductivity increases steadily with temperature up to 120 °C, reflecting enhanced proton transport at elevated temperatures. This behavior is consistent with increased proton mobility and polymer segmental dynamics within hydrated ionic domains [21]. The conductivity enhancement is closely linked to the water-retention characteristics revealed by TGA analysis (Section 3.6). Under low-humidity conditions, the ability to retain bound water becomes a decisive factor for sustaining proton conduction. Incorporation of SiO_2_ strengthens water binding within the membrane matrix through interfacial hydrogen bonding and structural confinement, thereby stabilizing hydrated proton-conducting pathways and mitigating dehydration-induced transport limitations.

All membranes exhibit a maximum proton conductivity at 120 °C. Among the composites, Nafion/SiO_2_-3 shows the highest conductivity, indicating that an optimal SiO_2_ loading effectively enhances water retention while preserving the continuity of ionic transport channels. At this composition, the dispersed inorganic phase forms a stable inorganic–polymer framework that supports efficient proton transport. Notably, the Nafion/SiO_2_-3 membrane exhibits a proton conductivity of 61.9 mS/cm at 120 °C and 50% RH, which is higher than or comparable to those reported for Nafion-based composite membranes under similar operating conditions, highlighting the effectiveness of the present sol–gel-assisted approach in improving proton conduction at elevated temperatures. At higher temperatures (130–140 °C), proton conductivity decreases markedly for all samples, which is attributed to progressive dehydration and thermally induced disruption of ionic domains. Despite this decline, the composite membranes consistently outperform pristine Nafion, underscoring the stabilizing role of SiO_2_ in maintaining proton transport under elevated-temperature conditions. By contrast, membranes with higher SiO_2_ loadings (4 and 5 wt%) exhibit persistently lower conductivity across the investigated temperature range. This behavior is likely associated with particle aggregation at excessive filler contents, which disrupts the continuity of sulfonic-acid-mediated proton transport pathways and introduces transport heterogeneity within the polymer matrix.

Together, these results demonstrate that precise control of SiO_2_ content is critical for balancing water retention, structural stability, and proton transport efficiency, enabling improved membrane performance under high-temperature and low-humidity operating conditions.

### 3.8. Single-Cell Fuel Cell Performance

**Figure 9:**
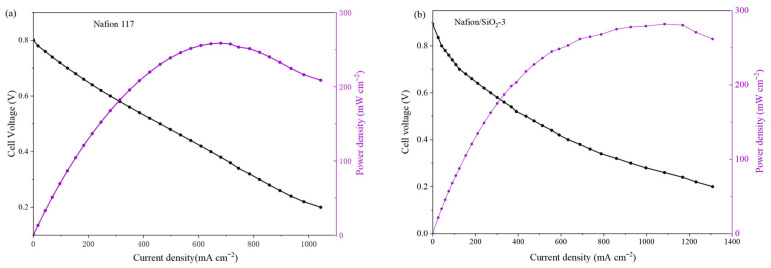
Single-cell polarization and power density curves of (**a**) Nafion 117 and (**b**) Nafion/SiO_2_-3 at 120 °C and 50% RH.

To evaluate whether the membrane-level improvements translate into practical device performance under MHT operation, single-cell tests were conducted using MEAs fabricated under identical procedures and tested under the same operating conditions. Figure 9 compares the polarization and power density curves of the reference Nafion 117 membrane and the Nafion/SiO_2_-3 composite membrane at 120 °C and 50% RH. Under these conditions, the Nafion/SiO_2_-3 MEA exhibits a higher open-circuit voltage (0.89 V vs. 0.8 V) and delivers improved output in the kinetically/ohmically dominated region, leading to a higher peak power density (282 mW cm^−2^ vs. 259 mW cm^−2^). Notably, the voltage decay of the composite-membrane MEA becomes less severe at elevated current densities, indicating better tolerance to dehydration and a delayed onset of performance losses under high-temperature, reduced-humidity operation. The performance enhancement observed at 120 °C is consistent with the membrane-level improvements in hydration retention and proton transport, which help mitigate dehydration-induced ohmic losses at elevated temperatures. Polarization curves measured at 80 °C and 100 °C under 50% RH are provided in the Supporting Information (Figure S5), where the performance differences between membranes are less pronounced under less demanding operating conditions.

It should be noted that the present MEA fabrication and operating parameters were not systematically optimized for achieving maximum absolute performance. Accordingly, the single-cell results presented here are intended as a proof-of-concept demonstration that the membrane modification can produce measurable benefits at the device level under 120 °C/50% RH operation, rather than as a direct benchmark against literature-reported state-of-the-art peak power densities obtained under different cell hardware, catalyst layers, and operating protocols. Future work will focus on separating the contributions of ohmic, charge-transfer, and mass-transport losses (e.g., via high-frequency resistance analysis and electrochemical impedance spectroscopy), as well as on optimizing MEA processing to fully leverage the high-temperature advantages of the composite membrane.

## 4. Conclusions

In this study, Nafion/SiO_2_ composite membranes with systematically varied SiO_2_ loadings were developed to address the intrinsic dehydration and conductivity limitations of PFSA membranes under MHT-PEMFC operation. Comprehensive structural and physicochemical analyses reveal that SiO_2_ nanoparticles are homogeneously dispersed within the Nafion matrix and form strong interfacial hydrogen-bonding interactions with sulfonic acid groups, leading to suppressed crystallinity, enhanced mechanical reinforcement, and stabilized hydrated ionic domains. Thermogravimetric analysis confirms that SiO_2_ incorporation significantly improves water retention in the temperature range relevant to MHT-PEMFC operation. Proton conductivity measurements at 50% relative humidity demonstrate that Nafion/SiO_2_-3 exhibits optimal transport performance, achieving the highest conductivity at 120 °C while maintaining superior conductivity retention at elevated temperatures compared to pristine Nafion. In addition, moderate SiO_2_ incorporation provides mechanical reinforcement without compromising membrane integrity, while excessive filler loading leads to embrittlement, highlighting the importance of compositional optimization. Beyond membrane-level characterization, a proof-of-concept single-cell evaluation at 120 °C and 50% RH further corroborates that the hydration/transport advantages of Nafion/SiO_2_-3 can translate into improved fuel-cell performance under identical MEA fabrication and operating conditions. Overall, this work establishes a clear structure–property relationship for SiO_2_-reinforced PFSA membranes and demonstrates that controlled inorganic incorporation offers a viable and scalable strategy for extending the operational temperature window of PEMFCs without sacrificing membrane integrity or durability.

## Figures and Tables

**Table 1:** Compositions of Nafion/SiO_2_ composite membranes prepared in this study.

Membrane Name	SiO_2_ Content (wt%)	Final Thickness (μm)
Nafion/SiO_2_-0	0	90 ± 5
Nafion/SiO_2_-1	1	92 ± 3
Nafion/SiO_2_-2	2	94 ± 3
Nafion/SiO_2_-3	3	96 ± 3
Nafion/SiO_2_-4	4	96 ± 6
Nafion/SiO_2_-5	5	100 ± 5

## Data Availability

Data will be made available on request.

## Supplementary Materials

The following supporting information can be downloaded at: https://www.mdpi.com/article/10.3390/polym18030329/s1, Figure S1: AFM height images of Nafion/SiO_2_ composite membranes with (a) 1 wt%, (b) 2 wt%, (c) 3 wt%, (d) 4 wt% and (e) 5 wt% SiO_2_; Figure S2: Large-area AFM height image (10 μm × 10 μm) of the Nafion/SiO_2_–5 composite membrane; Figure S3: XRD patterns of Nafion/SiO_2_ composite membranes with varying SiO_2_ contents measured at (a) 100 °C, (b) 120 °C and (c) 140 °C; Figure S4: Derivative thermogravimetric (DTG) curves of Nafion/SiO_2_ composite membranes; Figure S5: Single-cell polarization and power density curves for Nafion 117 and Nafion/SiO_2_-3 at 100 °C and 80 °C with 50% RH; Table S1: Surface roughness parameters (Ra and Rq) of Nafion/SiO_2_ composite membranes measured by AFM.

## Abbreviations

The following abbreviations are used in this manuscript:

PEMFC	Proton exchange membrane fuel cell
MHT	Medium- to high-temperature
PFSA	Perfluorosulfonic acid
RH	Relative humidity
CHP	Combined heat and power
PEM	Proton exchange membranes
AFM	Atomic force microscope
XPS	X-ray photoelectron spectroscopy
FTIR	Fourier transform infrared
XRD	X-ray diffraction
DMA	Dynamic mechanical analyzer
EIS	Electrochemical impedance spectroscopy
CCM	Catalyst-coated membrane
MEA	Membrane electrode assemblies
