# Supplementary material for: Enhancing Hydration Stability and Proton Transport in Nafion/SiO2 Membranes for Medium- to High-Temperature PEMFCs
Source: Polymers (Basel). 2026 Jan 26;18(3):329. doi: 10.3390/polym18030329 (PMC12899464; doi:10.3390/polym18030329)
Supplement: Supplementary file 1 [file polymers-18-00329-s001.zip › polymers-4102784-supplementary.pdf]

Support Information

Enhancing Hydration Stability and Proton Transport in Nafion/SiO_2 Membranes for Medium- to High-Temperature PEMFCs

Shuai Quan [1], Zheng Sun [1], Cong Feng [1,*], Lei Xing [2,*], and Pingwen Ming [3]

[1] College of Materials Science and Engineering, Tongji University, Shanghai 201804, China
[2] School of Chemistry & Chemical Engineering, University of Surrey, Guildford GU27XH, UK
[3] School of Automotive Studies, Tongji University, Shanghai 201804, China; pwming@tongji.edu.cn
* Correspondence: fengcong@tongji.edu.cn (C.F.); l.xing@surrey.ac.uk (L.X.)

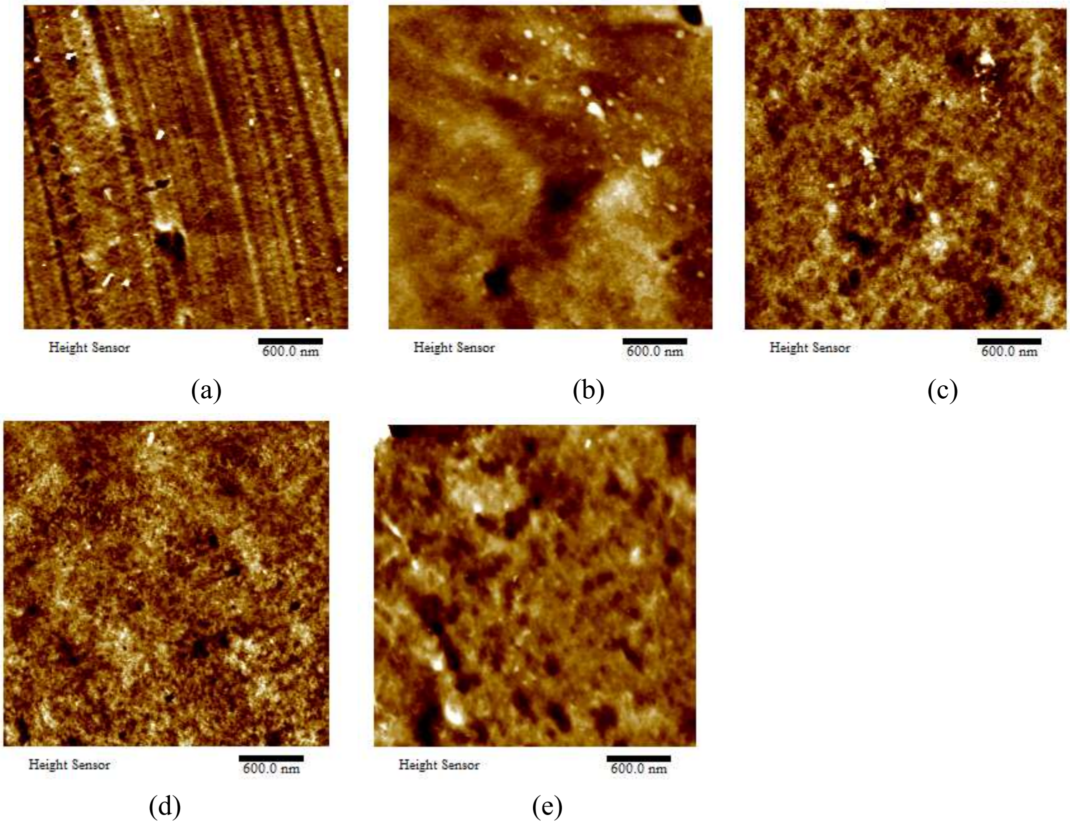

Figure S1. AFM height images of Nafion/SiO_2 composite membranes with (a) 1 wt%, (b) 2 wt%, (c) 3 wt%, (d) 4 wt% and (e) 5 wt% SiO_2

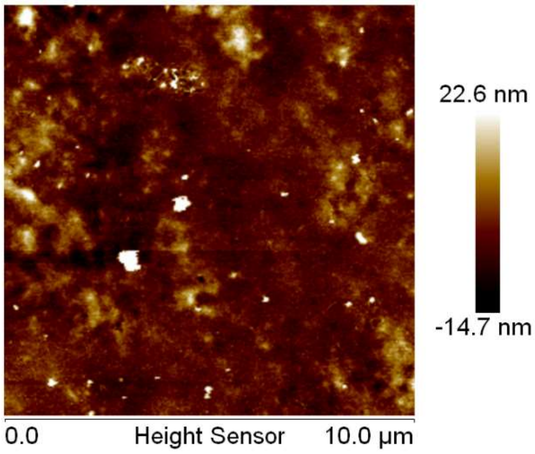

Figure S2. Large-area AFM height image (10 μm × 10 μm) of the Nafion/SiO_2–5 composite membrane. The large scan size is comparable to typical SEM observation scales and allows evaluation of surface morphology and possible nanoparticle agglomeration. No pronounced large-scale aggregation is observed, even at the highest SiO_2 loading.

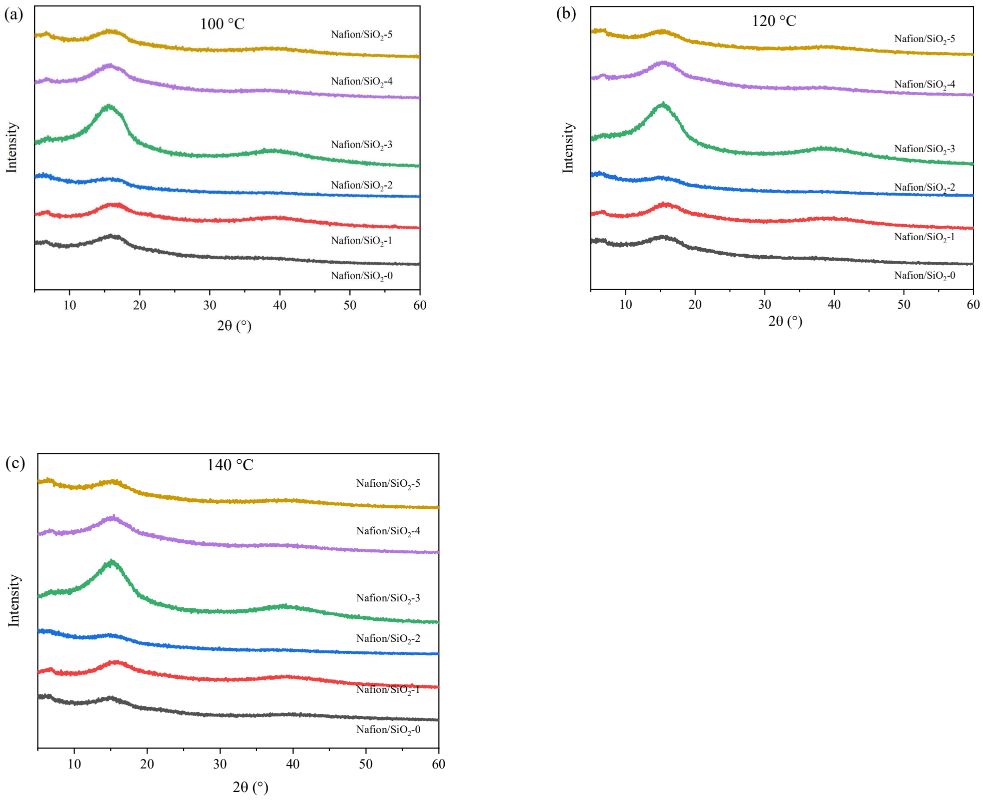

Figure S3. XRD patterns of Nafion/SiO_2 composite membranes with varying SiO_2 contents measured at (a) 100 °C, (b) 120 °C and (c) 140 °C.

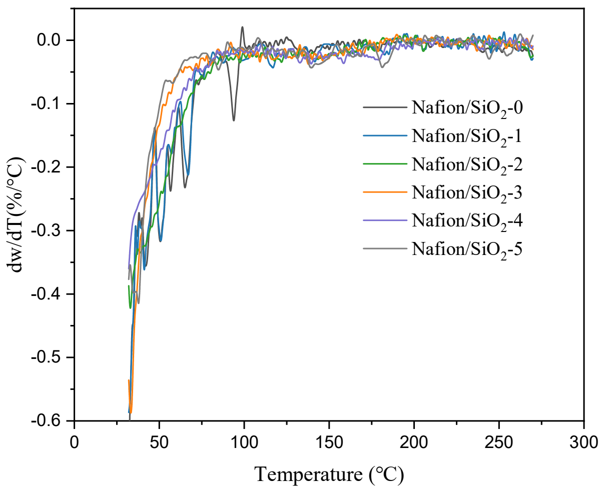

Figure S4. Derivative thermogravimetric (DTG) curves of Nafion/SiO_2 composite membranes. The DTG curves show minor weight-loss events at low temperatures, mainly associated with water removal, while no pronounced degradation peaks are observed up to 270 °C, confirming sufficient thermal stability within the investigated temperature range.

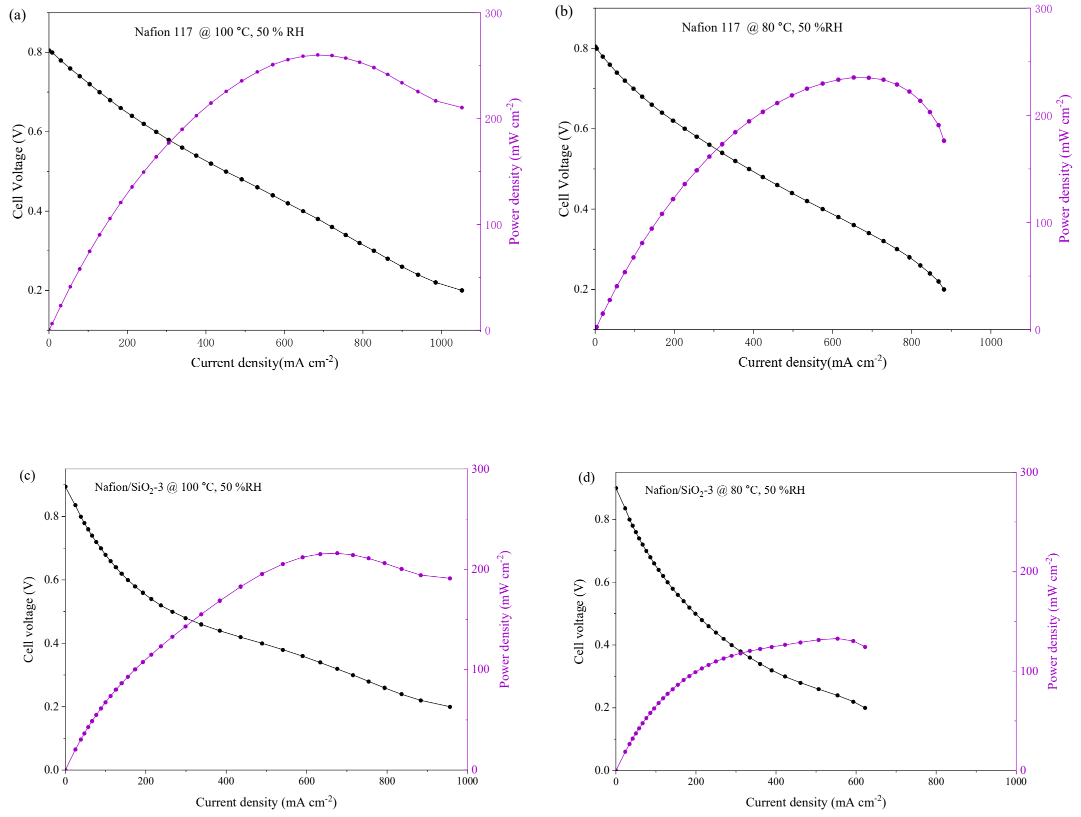

Figure S5. Single-cell polarization and power density curves for (a) Nafion 117 at 100 °C and 50% RH, (b) Nafion 117 at 80 °C and 50% RH, (c) Nafion/SiO_2-3 at 100 °C and 50% RH, and (d) Nafion/SiO_2-3 at 80 °C and 50% RH.

Table S1. Surface roughness parameters (Ra and Rq) of Nafion/SiO_2 composite membranes measured by AFM[1].

Membranes	Ra/nm	Rq/nm
Nafion/SiO_2-1	0.74±0.19	0.99±0.21
Nafion/SiO_2-2	1.57±0.32	2.35±0.62
Nafion/SiO_2-3	2.95±0.95	3.98±1.44
Nafion/SiO_2-4	1.26±0.65	1.89±1.20
Nafion/SiO_2-5	3.39±0.96	4.55±1.49

[1]The reported Ra and Rq values represent the mean ± standard deviation obtained from AFM measurements at five different locations on each membrane.
